# On Some Sources of Fallacy in the Diagnosis of Phthisis

**Published:** 1868

**Authors:** W. F. Wade

**Affiliations:** Physician to the General Hospital, Birmingham


On Some Sources of Fallacy in the Diagnosis of Phthisis. By W. F. Wade, B. A. M. B., Physician to the General Hospital, Birmingham.

In three-and-twenty years I have seen many cases in which mistakes have been made as to the diagnosis of phthisis, and I do not wish to pretend that all the mistakes have been in the practice of other persons, none in my own. It has long seemed to be one of the weakest points of our profession, that we have been in error; and there is, of course, a still greater reluctance to allow that the error has been an avoidable one. I have sometimes perhaps received credit for making a correct diagnosis in difficult cases, where, perchance, others had been less successful; but I have ever felt that my judment has been more improved, and my opinion, *quantum valeat*, rendered more valuable, by a candid and careful reflection upon cases where I have been mistaken, tnan by brooding on others of a more fortunate type. And yet we are all too apt to dwell upon our successes, and to ignore or unfairly extenuate our failures, even to ourselves. I say even to ourselves, for so long as we do not deceive ourselves, I do not say that it is necessary to parade our blunders before ignorant or unthinking persons; but, on the other hand, my experience has not taught me that wise and thoughtful people think the worse of one who is willing candidly and thoughtfully to admit an error, without seeking to gloss it over or palliate it in a disingenuous manner.

Regarding especially the errors made in the diagnosis of phthisis, we find that, as usual, they resolve themselves into those of omission and those of commission. A person is said to have phthisis whom the event proves to have been free from it; or, on the other hand, is pronounced free from it when, by the inexorable logic of subsequent facts, that opinion is falsified.

Before entering into special suggestions applicable to individual cases, it is opportune to point out one general source of fallacy applicable to all.

There is a general reluctance to admit that a case is doubtful, even when doubt is not merely the most philosophical, but the only philosophical, position. The general practitioner fears to imperil his hold upon the confidence of his patient by admitting that he cannot tell whether his patient is or is not phthisical. The consultant, called in for the express purpose of solving an admitted difficulty, requires still greater philosophical courage to confess that, whilst the evidence obtainable cannot fail to raise suspicion, it is yet insufficient to justify a positive opinion either one way or the other. And yet there is a stage in all organic diseases where we are justified only in doubting. And I entertain a conviction, which I trust may never be overturned, that in such cases, both as regards the profession and the public, honesty is, in the long run, the best policy. This position is true, and not only as regards those local organic diseases which are but the expression of constitutional conditions. For example, let us admit, not only that there is in phthisis a pre-tubercular stage, but that its peculiarities are such as to enable us to identify it prior to any development of its local expression. Even here we are compelled to admit that there is a time when it fails to afford sufficient evidence to justify an unqualified and unhesitating verdict, however shrewd and grave may be our suspicions.

There is, to my mind, but little practical inconvenience in having our suspicions aroused at a stage where we are unable to set them at rest. This is the time when, of all others, the case is remediable. It ought to be, and indeed I think is, admitted to be an axiom in medicine, that the patient should have the benefit of any doubt which may exist; and it will, I think, be admitted without discussion, that, in the vast majority of cases, the patient has that benefit if we advise such a course as we should do had circumstances compelled us to form a more positive though less favorable opinion. In all probability, such persons are broken down in health from over-lactation, overwork, or other exhausting causes; and the hygienic measures which are suitable to a case of early phthisis are just those which are most likely to relieve these conditions, which, if unattended to, are well calculated to produce either phthisis itself or a state of permanent ill-health scarcely more hopeful, and perhaps even more inconsistent with the active duties of life. Possibly the patient, less anxious, because less far-seeing than ourselves, may neglect our advice, and get well in spite of his neglect. We should feel satisfied if, upon a careful review of the case, we still feel that our advice was the most prudent possible. According to a very astute man, Talleyrand, it sometimes shows better judgment to be deceived than not to be deceived. Paradoxical as such a dictum may appear, the philosophy of it is not far to seek or difficult to understand. With regard to giving patients the advantage of a doubt, there is one other remark to make. The propriety of doing so is well illustrated in the kind of cases already alluded to, and from a consideration of them and others, we may deduce a very useful general law, viz: when in our diagnosis we halt between two opinions—one that the disease is a curable one, the other that it is incurable—we should treat the patient upon the hypothesis that his disease is curable. For example, we may doubt in one case between phthisis in some form and an idiopathic fever, or in another between simple gastric ulcer and cancer of the stomach. In the first instance, we should undoubtedly proceed upon the supposition that the case is one of idiopathic fever, and in the other that it is one of simple gastric ulcer. It seems to me that the reasons for this law are so obvious that it is unnecessary to specify them in detail.

Coming more particularly to the subject of this paper, it is scarcely necessary to remark that the general principles of the diagnosis of phthisis, both physically and symptomatically, are well established, and that I have therefore no intentioa to recapitulate or reorganize them. And it should be borne in mind that there is a wide difference between difficulties and fallacies. There will always remain cases in which, from one cause or another, the best informed physician, the most careful investigator, and the most wary man, will find great, perhaps insurmountable difficulties. But as regards fallacies, to be aware of them is to be able to avoid them. The case which is obscured by a source of fallacy of which we are ignorant becomes transparent the moment that source of fallacy is pointed out. It has thus occurred to me, from time to time, that certain minutiæ which by times, or rather the neglect of which by times, lead to error, might be usefully pointed out, and this I purpose doing with special reference to errors that have come actually under my notice.

Errors have been committed even by high authorities—some due unquestionably to simple carelessness, and others absolutely inexplicable—and to such I shall not further allude.

Amongst the sources of fallacy which beset the diagnosis of phthisis, pleurisy takes a foremost place. The relations of the two are, indeed, exceedingly complex and interesting; but it would be beyond the scope of this paper to regard them all.

A local dry pleurisy at the apex of the lung not unfrequently furnishes sounds which are with difficulty distinguished from the sounds, creaking or quasi-moist, dependent upon tubercular deposition. It is of high importance to distinguish between the two—of the more importance inasmuch as there are not a few practitioners who would jump to the conclusion that the pleurisy is a sufficient indication of tubercle in the adjacent lung. Careful observation has convinced me that local apicial pleurisy may effect a person with unquestionable tubercular tendency without being conjoined with any actual deposit of tubercle recognizable after the cure of the pleurisy.

Acute pleurisy with effusion produces at a certain stage accoustic phenomena, which physical examination, if limited to the subclavian space, cannot with any certainty distinguish from those of softened tubercle. The lung is pressed upward by the effusion, and so condensed as to afford breathing of a tubular character, to be distinguished from tubercular consolidation, or even from a cavity, with difficulty, if at all. The sounds of the voice and the vocal vibration lend us no assistance in differentiating the two conditions. Furthermore, the attrition of the parietal and visceral pleuræ, covered possibly with soft lymph and lubricated by the liquid effusion, produce sounds so similar to mucous rattles that they are calculated to deceive the most practised ear. We thus get an apparent combination of those phenomena which, according to Barth and Roger, (whose dictim on this point I am much disposed to accept), are the only sure diagnostics of a cavity—namely, tubular breathing with moist rattles at the same point. This fallacy is to be avoided by attention to the history of the case, by examination of the whole of the affected side, and not of the apex only, and more especially by a continued observation of the mutations of sound consequent upon progressive absorption of the fluid. At the same time it is to be noted that in such a case there may be a history which suggests tubercular deposit antecedent to the acute pleurisy. In such a case we are not justified in giving any opinion founded on physical examination till the pleuritic condition has absolutely passed away. The secondary changes which a lung in some cases undergoes in consequence of a severe pleurisy give rise to many physical phenomena, as well as rational symptoms, resembling those dependent upon tubercular deposit and softening. These have been carefully observed and well described by competent authorities; it is therefore unnecessary to say more here than that a want of due acquaintance with the literature of this most important topic has, more than once within my own knowledge, led to an erroneous diagnosis of phthisis. It may be useful just to note that in this condition the "cracked pot sound" is not unfrequently exceedingly well marked. This sound is also oftentimes to be produced by percussing the chest of children in whom there is no evidence, scarcely even a suspicion, of anything besides bronchitis; and in them it is often to be heard over a very considerable area.

It is believed by some persons, whose opinion is entitled to respect, that a dry pleurisy on the right side is most commonly indicative of, or at least associated with phthisis; and if it be so, it is a matter of practical importance.

What is called laryngeal phthisis is usually, if not invariably, ordinary pulmonary phthisis, with laryngeal complication. It is a disease commonly of great obscurity; that is to say, whilst the rational symptoms of phthisis are easily to be perceived, yet the stridor developed in the larynx in a great measure, often completely, obliterates the sounds produced by the pulmonary deposit. Now, it is a well-ascertained fact, though not so readily explicable, that in laryngeal phthisis it is the right lung which is affected, or, if both be diseased, that which is most deeply implicated. In such a case, then, the fact of a dry pleurisy on the right side is a valuable corroboration of our suspicions. Though not directly connected with pleurisy, it is convenient to refer here to another consideration which should weigh with us in the diagnosis of laryngeal phthisis. Inasmuch as the lung disease, if any be present, will be found in the vast majority of instances, on the right side, it is clear that any suspicious or indefinite sound heard on the right side is vastly more significant than if it occurred on the left. It is not needful to specify more minutely the various sounds which come within the category of suspicious ones; they are sufficiently familiar to any one who takes an interest in this question.

The physical signs of a pneumonia at the apex might readily be mistaken for those of tuberculous deposit by an observer who neglected to take into consideration the history of his patient. It is true of pneumonia as of pleurisy at the apex, that during its presence we must positively decline to give a decided opinion that tubercle is absent—that is to say, unless we have had an opportunity of making special investigation prior to the pneumonic attack. At the same time it is, I think, quite an exception to meet with distinct physical evidence of apicial pneumonia in a tuberculous lung. And for my own part, I should consider that unquestionable pneumonia of the apex was, as far as it went, an encouragement to believe that the apex was free from tubercle.

Had it not occurred to me to see both mistakes made, I should scarcely have ventured to imagine that phthisis might be mistaken for typhoid fever, and typhoid fever for phthisis. In expressing surprise at this, I should have said that the former mistake was not made in a case of acute phthisis, the diagnosis of which is (at a certain stage, at least,) one of much difficulty. Were practitioners habitually careful, when dealing with any case of pyrexia, to consider, in the first instance, whether the fever belonged to the great class of primary fevers or to the other great class of secondary fevers, such a mistake could scarcely be made by any moderately competent person.

We now turn to a consideration of some circumstances which may lead to a fallacious opinion thot phthisis is absent when it really exists.

In the first place, it must be borne in mind that there is no pathognomonic physical sign of any stage or condition of tubercular deposit in the lungs. The process by which we arrive at the diagnosis of phthisis is a reasoning one of some complexity. It is true that in the ordinary diagnosis of flagrant cases several members of this argument are suppressed. Indeed, so commonly is this done that many persons never know how many or what links there really are in the chain. But a want of this knowledge is certain, on some occasions, to lead to avoidable error. Imperfect as our science of diagnosis must be admitted to be, I see more mistakes made through the imperfection of the individual using it than through the imperfection of the science itself. I cannot avoid remarking here that formal logic is often most derided by those who have most need of it. And this contempt is often excused by reference to some of those quasi self-evident propositions which are given in illustration of the syllogistic form. It is just as if one were to sneer at arithmetic because the fact that two and two make four might be given as an illustration of one of its rules. There is no such test of the accuracy of our premises and the correctness of our conclusions as the reduction of an argument to the form of a syllogism. Were this done, how many things in medicine which are supposed by the mass to be proved would turn out to be merely probable in a higher or lower degree!

Some of the most important signs of phthisis, and, indeed, those of which we most commonly avail ourselves in practice, are moist sounds, rattles of various kinds at the apex of the lung. Connected with these are several sources of fallacy; and as these signs are those most often actually used, so these fallacies are those which most often lead astray. In the first place, moist sounds, of whatever kind, even at the apex of the lung, and there only, are *per se* no evidence of phthisis at all. Their value as evidence depends entirely upon the perfection of the chain of argument of which they are, it is true, a most valuable link. One peculiarity of these sounds, which careful persons do not neglect to ascertain, is their permanency. It is curious how, day after day, we may examine a chest and find always there the same click or the same mucous râle. They are not permanent because they are directly produced by tubercle, for they are not; but by mucous secretion, of which, indeed, the tubercle, which is permanent, is the immediate cause. This is very far from being a distinction without a difference. The truth is, that in not a few cases these, reputed permanent, râles occasionally disappear, and we then lose a very important element of our diagnosis. A little consideration of the causes of this occasional absence will enable us in many cases to secure the assistance which their presence really affords us.

In the first place, it often requires a full, free inspiration to develop them, even when the mucous is present in sufficient quantity. Thus, in feeble, stupid, or nervous persons, it may be impossible to obtain sufficient respiratory freedom unless their circulation and respiration are so quickened by exercise as to compel them to breathe freely, whether they will or no. It is clear that such persons should not be examined in bed only.

The formation of the upper portion of the chest in some persons causes so much murmur, from pressure on the subclavian by the stethoscope, as to be a real source of embarrassment and difficulty; but it can scarcely be termed with propriety a source of fallacy.

In another class of cases the removal of mucus from the chest, which, as we know, is common after rising in the morning, is so complete that none is left to produce a rattle. In such cases it is clear that we avoid a great source of fallacy by examining the patient in bed, before the morning expectoration has occurred. The practical lesson we draw from these two classes of cases is that, in doubtful cases, where the history, symptoms, or appearance of the patient suggest the probability of latent phthisis, we should invariably abstain from pronouncing him to be free from it till we have had an opportunity of making careful examination under different, and indeed, as nearly as may be, opposite circumstances.

Another practical lesson of almost equal importance is that, in examining a case of suspected phthisis, we should listen to each side during ordinary respiration before making the patient take forced or deep inspirations; and again we should be careful, when the patient does take a forced inspiration, to listen first to the lung which we more strongly suspect to be the seat of disease, lest, while we are listening to the sound side, the forced respiration should have removed the mucus, and with it the morbid sounds it produces, from the diseased lung. On the other hand, if we hear a moist rattle at the apex, we must not forget that it is not impossible to have mucus there, and there only, in a non-tubercular lung. But then it is not likely to be there constantly, or even frequently.

It is principally from considerations of this kind that the wisdom of the following axiom becomes apparent—viz:

"Never pronounce an opinion upon a case of supposed phthisis without more than one examination."

There is another reason for observing the precautions above mentioned. I entertain no doubt that in some cases of early phthisis some auscultatory phenomena—for example, feeble inspiratory murmur, possibly also semi-bronchial breathing—depend upon collapse of the lung. This collapse is capable of being removed by forced respiration; and we may thus deprive ourselves of valuable information, which, if it did not justify us in pronouncing the case to be absolutely one of phthisis, would, at all events, guard us against the opposite danger.

The muscular murmur is certainly sometimes a source of fallacy. imitating, as it may do, abnormal respiratory sounds; it is also a source of fallacy in the diagnosis of heart disease. In the case of the lungs, we should be careful to listen attentively to the muscular murmur whilst the patient abstains from breathing; we shall by that means be able to estimate its real amount, and we shall not afterwards find much difficulty in disentangling it, if I may use the expression, from the genuine breath sounds.

The information afforded by auscultation and percussion is so precise, and commonly so full, that the necessity for occasionally supplementing it by other means is so generally recognised as it ought to be. The thermometer has of late been made serviceable in this respect.

In acute phthisis the stethoscopical phenomena are inadequate to establish the diagnosis, at all events for some time. I have consequently known of instances where the practised eye of a physician unskilled in the use of the stethoscope has recognised the true nature of the case, which a skilled stethoscopist, relying solely on his instrument, has failed to do. It is scarcely needful for me to say that I have no desire to depreciate physical diagnosis, but only to point out that in this special class of phthisis it is a source of fallacy if it be too exclusively relied upon.

In some cases of senile phthisis, too, from the general diffusion of the tubercle, the stethoscopical signs are chiefly those of bronchitis, and an implicit reliance upon them leads to error.

In such cases as these the thermometer is a more reliable guide than the stethoscope. But it must not be forgotten that it too requires to be read by an instructed mind, and not merely by the eye. False inferences may be deduced from thermometrical as from other observations. The following is an illustration. A man was found during convalescence from small pox to have an unduly high temperature, without evident cause; the inference of tuberculosis was drawn. He left the hospital, and passed from under notice. About twelve months after he was seized with symptoms of paralysis, and admitted into hospital under my care. He shortly became comatose, and died. On dissection we found a chronic abcess of the brain, and one also of the spleen, which was, indeed, converted into a bag of pus. His body was quite free from tubercle. It is well known that before we draw from the temperature any inference with regard to tubercle, we must first of all exclude, amongst other things, the possibility of local suppuration. Whether the error in this case was avoidable or not I have no means of judging. If it was not, it points out a source of fallacy in the thermometrical diagnosis of phthisis. It is of importance to note briefly that some cases of cancer of the lung are with difficulty to be distinguished from phthisis, and that as there is such a thing as cancerous hectic, a too exclusive reliance upon the thermometer may lead us widely astray, as I have in one instance found.

The microscope has lately been made more available for the detection of phthisis, and it will afford us invaluable assistance in cases where the stethoscope fails. For example, in cases of what is called "latent pleurisy," no amount of purely stethoscopical skill will avail us; the physical signs of pleurisy drown those of phthisis. Indeed, I think that an expert stethoscopist, trusting, as there is danger of doing, too implicitly to his ear, is more likely to be deceived than a less skilled person trusting to his common sense, and judging from the general appearance and history of the patient.

It has long been known that in phthisis the lung is disintegrated, and that portions of it are expectorated. It has also been long known that these fragments of lung-tissue can be identified by the microscope. But the difficulty of discovering them, even in cases where he might be certain that they were present, from their being inextricably mixed up with mucus and pus, has prevented the microscopical diagnosis of phthisis being as much employed as it ought to have been.

We are, therefore, deeply indebted to Dr. Fenwick, who has suggested a plan of treating the expectoration, which enables us to find the fragments of lung-tissue, and place them in the field of the microscope with as little trouble as it costs us to demonstrate tube-casts in the urins. This facility of manipulation will doubtless lead to a much more frequent employment of this means of supplementing and correcting the information derivable from other sources in cases of supposed phthisis. We shall probably before long have an opportunity of ascertaining from a multiplicity of observations the exact value of this particular method of investigation. That it, like the stethoscope and the thermometer, has its own peculiar sources of fallacy we may well expect to find. If so, it will the better teach us the necessity of correcting one kind of evidence by another, and that accuracy of diagnosis requires care in observation, caution in deduction, and withal modesty remembering that to err is human.—*London Lancet.*

BIRMINGHAM, Dec. 1867.

